# The Role of the Frank–Starling Law in the Transduction of Cellular Work to Whole Organ Pump Function: A Computational Modeling Analysis

**DOI:** 10.1371/journal.pcbi.1000371

**Published:** 2009-04-24

**Authors:** Steven A. Niederer, Nicolas P. Smith

**Affiliations:** Computing Laboratory, University of Oxford, Oxford, United Kingdom; University of Auckland, New Zealand

## Abstract

We have developed a multi-scale biophysical electromechanics model of the rat left ventricle at room temperature. This model has been applied to investigate the relative roles of cellular scale length dependent regulators of tension generation on the transduction of work from the cell to whole organ pump function. Specifically, the role of the length dependent Ca^2+^ sensitivity of tension (Ca_50_), filament overlap tension dependence, velocity dependence of tension, and tension dependent binding of Ca^2+^ to Troponin C on metrics of efficient transduction of work and stress and strain homogeneity were predicted by performing simulations in the absence of each of these feedback mechanisms. The length dependent Ca_50_ and the filament overlap, which make up the Frank-Starling Law, were found to be the two dominant regulators of the efficient transduction of work. Analyzing the fiber velocity field in the absence of the Frank-Starling mechanisms showed that the decreased efficiency in the transduction of work in the absence of filament overlap effects was caused by increased post systolic shortening, whereas the decreased efficiency in the absence of length dependent Ca_50_ was caused by an inversion in the regional distribution of strain.

## Author Summary

The heart achieves an efficient coordinated contraction via a complex web of feedback loops that span multiple spatial and temporal scales. Advances in computational hardware and numerical techniques now allow us to begin to analyse this feedback system through the use of computational models. Applying this approach, we have integrated a wide range of experimental data into a common and consistent modelling framework representing the cardiac electrical and mechanical systems. We have used this model to investigate how feedback loops regulate heart contraction. These results show that feedback from muscle length on tension generation at the cellular level is an important control mechanism of the efficiency with which the heart muscle contracts at the whole organ level. In addition to testing this specific hypothesis, the model developed in this study provides a framework for extending this work to investigating important pathological conditions such as heart failure and ischemic heart disease.

## Introduction

Contraction of the heart is a fundamental whole organ phenomenon driven by cellular mechanisms. With each beat the myocytes in the heart generate tension and relax. This local cellular scale tension is transduced into a coordinated global whole heart deformation resulting in an effective, organized and efficient system level pump function. Fundamental to achieving this efficient transudation of work is the integration of organ, tissue and cellular scale mechanisms. However, while efficiency is important in the heart, the role and relative importance of the underlying mechanisms responsible for achieving the efficient transduction of work from the cell to the organ (ETW) remains unclear.

In the healthy heart, structural heterogeneities in the morphology, electrophysiology, metabolic and neural mechanisms provide a stable physiological framework that facilitates a coordinated contraction [1] resulting in the ETW. However, over shorter time scales, sub cellular mechanisms are the most likely candidates for regulating the ETW in the face of dynamic variation in cardiac demand. Specifically, the sarcomeres themselves contain tension and deformation feedback (TDF) mechanisms that regulate the development of active tension based on the local tension, strain and strain rate. These provide a regulatory process to modulate deformation and tension signals experienced by the cell into a coordinated global response [2]–[4].

The four major TDF mechanisms are (1) length dependent changes in Ca^2+^ sensitivity (Ca_50_) [5] , (2) filament overlap [6], (3) tension dependent binding of Ca^2+^ to troponin C (TnC) [7] and (4) velocity dependent cross bridge kinetics [8]. TDF mechanisms 1 and 2 are characterised by the length dependent changes in the steady state force Ca^2+^ relationship, which is routinely described by a Hill curve [5],[9]. Length dependent changes in Ca_50_ are measured by the decreased concentration of Ca^2+^ required to produce half maximal activation as the muscle increases in length. Length dependent changes in the filament overlap result in active tension increasing as the muscle increases in length. Ca^2+^ binding to TnC acts as a switch activating tension generation. As crossbridges bind to generate tension they increase the affinity of Ca^2+^ to TnC causing more Ca^2+^ to bind, which results in the generation of more tension. The velocity dependence of tension can be described by a transient and stable component. The transient component is characterised by the multiphase tension response to step changes in length and the stable component is characterised by the tension during contraction at a constant velocity. In general as the velocity of contraction increases the active tension decreases.

These four mechanisms provide both positive and negative feedback for tension development and are fundamental to the functioning of the heart, yet their relative roles, if any, in the ETW have not been investigated. This is in part due to the experimental challenges in studying subcellular function in whole heart preparations [10] and the modelling challenges in performing biophysical whole organ coupled electromechanics simulations [11],[12]. Recent advances in computer power and coupling methods [13] now allow the simulation of strongly coupled multi-scale electromechanical models of the left ventricle. These models contain explicit biophysical representations of cellular electrophysiology, Ca^2+^ dynamics, tension generation, deformation and the multiple feedback loops that operate between each of these systems.

In this study we analyse the transduction of local cellular scale work into whole organ pressure-volume work in the heart using computational modelling. Using the definitions of Hill [14] for positive (shortening) and negative (lengthening) work, we propose a new metric to quantify the ETW during each phase of the contraction cycle as the ratio of positive work to total work. To isolate and quantify the role of TDF in the transduction of cellular work into whole organ pump function over a heart beat we have developed a model of the rat left ventricle, at room temperature, that incorporates the TDF mechanisms. The model contains a biophysical electromechanical rat myocyte model [15], transversely isotropic constitutive law [16] and heterogeneous fiber orientation [17]. By comparing the ETW over each phase of the heart beat in the absence of each of the TDF mechanisms we aim to quantify the effect of each of the TDF mechanisms.

## Methods

The model developed in this study simulates a rat heart functioning at room temperature. This is the sole species and temperature combination in which it is currently viable to study complex coupled electromechanics phenomenon due to limited data in other species-temperature combinations. The heart model is described by the geometry and fiber structure, cell model, myocardium model and boundary conditions. The model is defined and solved within the CMISS (Continuum Mechanics, Image processing, Signal processing and System identification) software package, written in FORTRAN and developed at the University of Auckland (www.cmiss.org). The code was compiled using the INTEL FORTRAN compilers for Itanium CPUs and solved using 16 CPUs and 20 Gb RAM on the ORAC super computer at the University of Oxford. All visualizations are generated using the freely availably CMGUI graphical user interface.

### Ventricle geometry model

**Figure 1:**
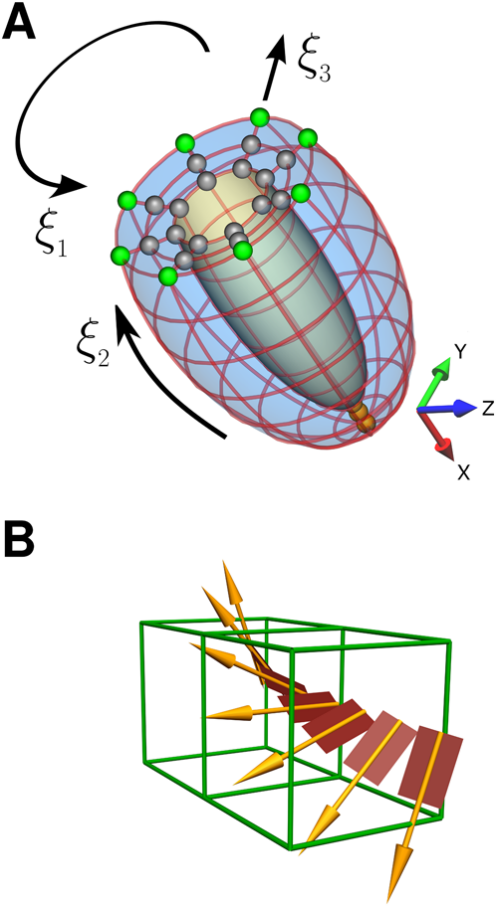
Model geometry, boundary conditions and fiber orientation. (A) Left ventricle geometry, boundary conditions are applied to the highlighted nodes as described in Table 2. Gray spheres are mid/inner nodes, green spheres are outer base nodes and orange spheres are the apex nodes. (B) Fiber and sheet orientation across the heart wall.

The rat left ventricle was approximated using a truncated ellipsoid (Fig. 1A). The mesh is described by tri cubic Hermite finite elements with an embedded fiber orientation, as described previously [18],[19]. The mesh consists of 195 nodes and 128 elements, with 8 elements in the circumferential, 8 elements in the base to apex and 2 elements in the transmural directions, totalling 4680 degrees of freedom. The heart is orientated with the apex to base axis aligned with the global *x* direction and the radial direction lies in the *y* and *z* plane, where the *x*, *y* and *z* directions are an orthogonal rectangular Cartesian co-ordinate system. The element co-ordinate system has ξ_1_ in the circumferential direction, ξ_2_ in the apex to base direction and ξ_3_ in the radial direction. The mesh dimensions were set to an endocardial and epicardial radius of 2.4 mm and 5.1 mm at the widest point and 13.2 mm and 11.5 mm from apex to base on the epicardium and endocardium, respectively [16],[20]. The resulting ellipsoid has an epicardial and endocardial ellipticity of 0.31 and 0.6, respectively and a cavity volume of 156 µL.

The fiber orientation describes the spatial variation of orthogonal axis aligned with the myocardium microstructure. The three directions that make up the axes correspond to the fiber, sheet and sheet normal directions. In the model heart the fiber orientation is described by three angles, named fiber, imbrication and sheet [21]. These field values are stored at each node and interpolated within the material space of the finite element mesh using tri linear basis functions. In this study the fiber orientation is assumed to only vary in the transmural (ξ_3_) direction and is constant in the circumferential (ξ_1_) and apex-base (ξ_3_) directions. The orientation was determined using confocal imaging data [17] that measured the fiber orientation across a wedge of the rat left ventricle wall. The fibers varied transmurally from 70° to −70°, the imbrication angle was 0° at all points and the sheet angle varied from 61° to 55.5° to 50° at the epicardial, midwall and endocardial nodes, respectively, as shown in Fig. 1B.

### Cell model

Cardiac myocytes were modelled using our previously developed coupled electromechanics model of the rat ventricular myocyte at room temperature [15]. The model combines the Pandit [22] electrophysiology model and the Hinch [23] Ca^2+^ dynamics model with our model of contraction [24]. The original model [15] was developed specifically to capture electromechanical function and has been extensively validated against experimental results [15], [22]–[24]. This framework has been previously used to study the slow force response to stretch and includes both short term (changes in tension between beats) and long term (changes in tension over multiple beats) length, velocity and tension dependent regulators of tension development. Due to the computational load of the whole organ model, it was not possible to perform simulations over periods of minutes, so the long term regulators of tension under physiological conditions (stretch activated channels, pH regulation and RyR regulation, included in our original cellular study) were not included in this study. The initial conditions in all simulations were determined by pacing a single cell version of the model to a steady state over 3,000 beats at 0.5 Hz with a stimulus current of −0.04 µAmm^−2^ for 10 ms. The model is integrated using the adaptive, stiff LSODA solver with maximum time step of 0.01 ms.

### Mechanics model

**Figure 2:**
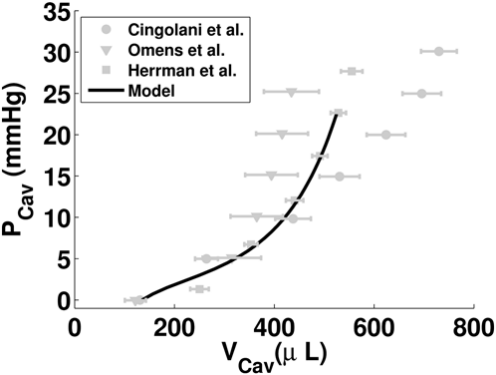
Validation of model (black solid line) passive pressure volume relationship compared with Cingolani et al., [31] (•), Omens et al., [16] (▿) and Herrmann et al., [32] (□).

**Figure 3:**
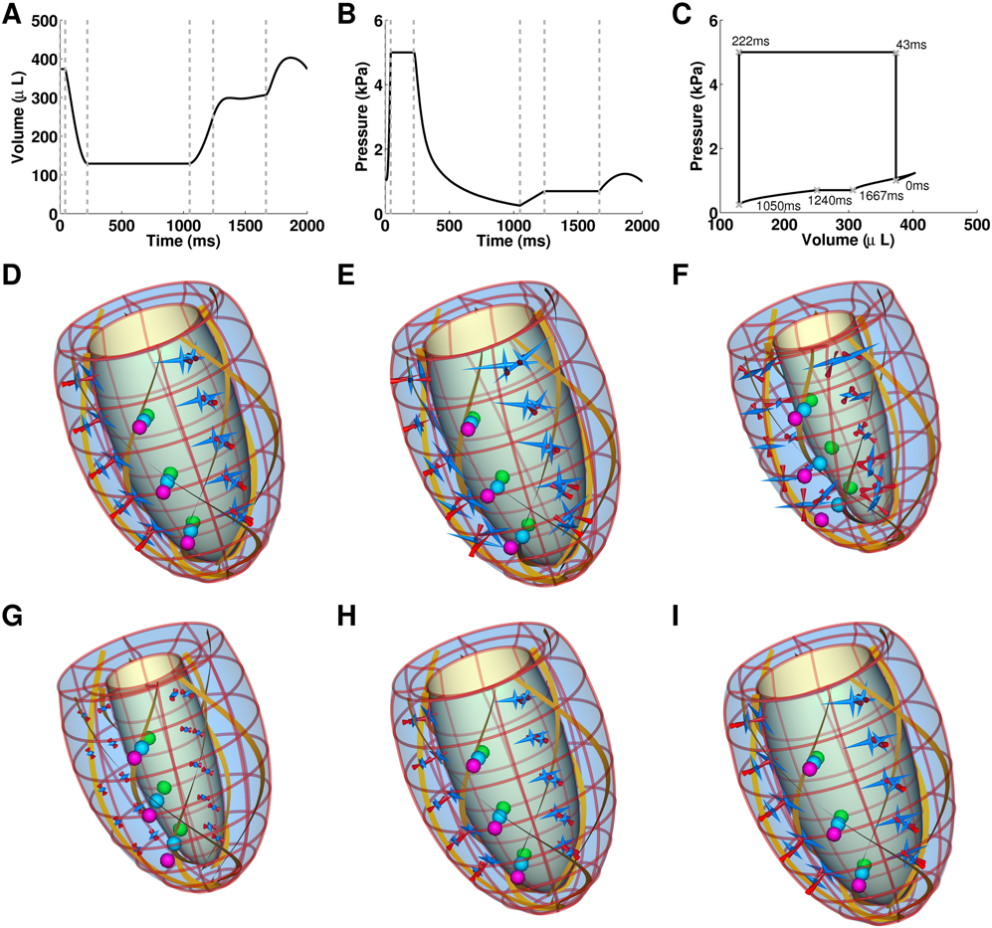
Normal model contraction cycle. Plots of (A) volume, (B) pressure and (C) P-V curve for the left ventricle model. Panels (D) to (I) show the ventricle model at (D) end diastole (0 ms), (E) end IVC (43 ms), (F) end ejection (222 ms), (G) end IVR (1050 ms), (H) end recoil (1240 ms) and (I) end diastases (1667 ms). The orientation and size of the cones embedded within the mesh indicate the direction and magnitude of principal strain, respectively. Blue and red cones represent states of compression and tension, respectively. Gold stream lines indicate the fiber orientation. The 3 coloured spheres assist in visualizing the rotation of the ellipsoid.

Deformation is described by the incompressible finite elasticity equations [25]. The heart is assumed to be in a quasi steady state, allowing the equations to be simplified by the removal of the inertia term. The constitutive equations are defined by a strain energy function given by,
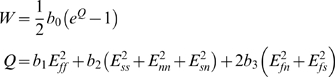
(1)where b_0_, b_1_, b_2_ and b_3_ are the model parameters, E_ff_, E_ss_ and E_nn_ are the Green strains in the fiber, sheet and sheet normal fiber axes directions and E_fn_, E_fs_ and E_sn_ are the Green shear strains in the fiber – sheet normal, fiber – sheet and sheet – sheet normal planes. The fiber, sheet and sheet normal directions correspond to the fiber axes aligned with the microstructure of the myocardium, described above. The constitutive law is based on a model of the rat myocardium developed by Omens et al. [16], who fitted the model parameters using a cylindrical approximation of the heart, thus ignoring any stiffness in the heart due to the apex. In this study an ellipsoidal approximation of the left ventricle geometry, containing an apex, is employed. To take account of any additional stiffness from the apex the scaling parameter b_o_ from the Omens model was refitted to allow the passive ventricle mechanics model to match passive pressure-volume relationships reported in the literature (see Fig. 2). The final parameters for the constitutive equation are listed in Table 1. The passive mechanics law is strongly coupled to the cellular active tension model as described previously [13]. Deformation and pressure are interpolated using tri-cubic Hermite and tri-linear basis functions, respectively. The equilibrium equations are integrated over each element using 3×3×3 Gaussian quadrature and this set of nonlinear equations are then solved using the Newton-Raphson method every 1 ms [26]. The mechanics model has fixed and temporal boundary conditions. The fixed boundary conditions prevent free body motion and constrain the base of the heart. Fig. 1A and Table 2 summarize the fixed boundary conditions. During the contraction cycle the boundary conditions on the cavity switch between defined pressure and volume. During isovolumetric contraction (IVC) and relaxation (IVR) the cavity volume is fixed. During ejection the pressure is fixed at 5 kPa (37.5 mmHg), maximizing the external work performed by the model (within 0.1 kPa). Ejection begins when pressure during IVC reaches 5 kPa (37.5 mmHg) and ends when the ventricle volume ceases to decrease. During diastole a transient cavity pressure was defined to represent initial filling, diastasis and the atrium contracting. The transient is taken from a Wiggers' plot [27] and is scaled to start at 0.25 kPa (1.88 mmHg), finish at 1 kPa (7.5 mmHg) and span the duration between end IVR and IVC (see Fig. 3B, below). Diastole begins when the pressure during IVR reaches 0.25 kPa (1.88 mmHg) and finishes when the next stimulus is applied.

**Table 1:** Coefficients of Omens material laws. b_0_ was fitted using passive inflation data.

Parameter	Value
b_0_	0.4
b_1_	9.2
b_2_	2.0
b_3_	3.7

**Table 2:** Boundary conditions defined with respect to global co-ordinate system and derivatives with respect to local ξ co-ordinates, where u_i_ is the displacement in the i^ith^ direction.

Node group	Deformation in i^th^ direction u_i_ set to zero
outer base nodes (Green sphere Figure 1)	u_x_, ∂u_x_/∂ξ_1_, ∂u_x_/∂ξ_3_, ∂^2^u_x_/∂ξ_1_∂ξ_3_ u_y_, ∂u_y_/∂ξ_1_, ∂u_y_/∂ξ_3_, ∂^2^u_y_/∂ξ_1_∂ξ_3_ u_z_, ∂u_z_/∂ξ_1_, ∂u_z_/∂ξ_3_, ∂^2^u_z_/∂ξ_1_∂ξ_3_
mid / inner base nodes (Gray spheres Figure 1)	u_x_, ∂u_x_/∂ξ_1_, ∂u_x_/∂ξ_3_, ∂^2^u_x_/∂ξ_1_∂ξ_3_
Apex nodes (orange spheres Figure 1)	all derivatives all directions

### Electrophysiology tissue model

Electrical propagation across the myocardium was simulated using the mono-domain approximation of the bi-domain equations [28]. The mono-domain equations were solved on a tri-linear finite element grid, embedded within the deforming material space of the whole organ geometry [29] with average grid spacing of 0.22 mm. The conductivity tensor is transversely isotropic with a conductivity of 0.263 mSmm^−1^ and 0.0262 mSmm^−1^ in the fiber and cross fiber directions, respectively [30]. The sensitivity of model simulations to the fiber conductivity is discussed below. An electrical stimulus of 100 µAmm^−3^ was applied to all cellular models located on the endocardial surface at 0.5 Hz for 10 ms to simulate the initiation of excitation by the Purkinje fiber network. The stimulus duration was longer than expected physiologically to insure a robust initiation of an activation wave. This protocol was chosen to ensure that the stimulation protocol was consistent across all simulations in the presence of physiological and non physiological changes to the cellular model. The impact of the stimulus duration and location on simulation results is estimated in the sensitivity analysis.

### Metrics of efficient work transduction and homogeneity

The ETW is quantified by a new metric η. The value of η relates the total amount of work performed by the cells to the amount available to pump blood around the body. As the heart converts cellular work into whole organ pump function best when the whole heart contracts and relaxes in unison, η also provides an active tension weighted indicator of cardiac synchrony. We also define the variability of tension and stretch, which provide metrics of homogeneity.

The total external work performed by the ventricle (*W_ext_*) is equal to the work done to eject blood from the ventricles, less the work done by blood returning to the ventricle and inflating it during diastolic filling. *W_ext_* is calculated using
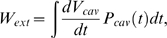
(2)where *V_cav_* and *P_cav_* are the cavity volume and pressure, respectively. As there are no viscous terms in the mechanics model the sum of the work performed by all the cells must be equal to the external work performed by the ventricle over a heart beat. A corollary of this statement is that the net work performed by the myocytes (*W_myo_*) over the myocardium (Ω) to contract the ventricle is equal to the *W_ext_*. *W_myo_* is calculated using
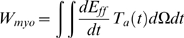
(3)where E_ff_ is the Green strain in the fiber direction and *T_a_* is the second Piola Kirchoff active tension tensor, which is assumed to only act in the fiber direction. The fiber direction component of T_a_ is calculated from the Cauchy stress active tension simulated by the cell model. For further background on modelling cardiac mechanics notation see Nash and Hunter [26]. The work that is performed by the myocytes can be either positive or negative, depending on the direction of the velocity. Positive work is produced by contracting cells and is converted into external work or stored as potential energy in the passive myocardium. Negative work results from stretching a tension generating myocyte and decreases the amount of myocyte positive work available for external work. Positive cellular work (W_pos_) and negative cellular work (W_neg_) are defined as
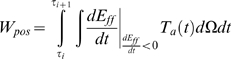
(4)and
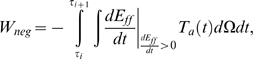
(5)respectively. The time integrals in Eq. 4 and 5 are between τ_i_ and τ_i+1_ which correspond to the start and end time of a phase of the contraction cycle. The myocytes consume energy in the form of ATP when they generate tension and this is true whether the myocytes are performing positive or negative work. We assume that the consumption of energy by the myocyte is the same for both positive and negative work, so that total cellular work is equal to the sum of the positive and negative work. This assumption is motivated by the limited cardiac muscle specific experimental data to quantitatively describe the relative consumption of energy for positive and negative work, the ramifications of this assumption are discussed below. Thus we define η as the ratio of cellular positive work to the total work (sum of the positive and negative work) performed by the myocytes. η is defined by
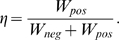
(6)


The form of η was chosen as it provides a simple, model independent, link between whole organ function and cellular work in the absence of a metabolic model component within the cell model to explicitly link the transduction of chemical energy to contractile function. High efficiency of work transduction, as measured by η, occurs when η approaches one during the contraction phases (IVC and ejection) and approaches zero during the relaxation phases (IVR and diastole). When η is one the whole ventricle is contracting and when η is zero the whole heart is relaxing, thus when the heart is efficiently converting work form the cells to pump function, it is also contracting synchronously.

As a contraction that efficiently converts cellular work to whole organ pump function coincides with a synchronous contraction we analyse the effect of TDF mechanisms on the spatial homogeneity of stress and strain at specific points in time. The homogeneity of contraction is quantified using a measure of variability, where the variability (θ) of an arbitrary variable (*a*) is calculated by normalizing the standard deviation (σ) by the median value (ω) (Eq. 9). The addition of a bar above a variable indicates the average.
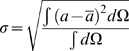
(7)

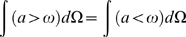
(8)

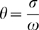
(9)


## Results

### Model validation

The model developed here is complex and represents a wide range of experimental results. The contraction [24], Ca^2+^ dynamics [23], electrophysiology [22] and the integrated cell model [15] been developed and validated previously. As such we have focused our model validations against whole heart measurements.

The passive material constitutive law was scaled to fit experimental pressure-volume (P-V) relationships recorded in quiescent rat hearts. The P-V relationship calculated by the passive heart model is compared with experimental results from Cingolani et al. [31], Omens et al. [16], and Herrmann et al. [32] in Fig. 2. The contracting heart model is solved to the mechanical steady state. The mechanical steady state was reached when the P-V loop returned to its initial state at the end of a beat, closing the P-V loop, as demonstrated in Fig. 3C. To determine how many beats were required to reach the mechanical steady state the model was run for six beats. After the first beat the model has reached the mechanical steady state. All further results are taken from the second beat following initialization, unless otherwise specified. Fig. 3A–C shows the pressure and volume traces for the normal heart model and Fig. 3D–I shows images of the heart model at the end of the six phases of contraction. The flat top of the pressure volume curve is consistent with rat P-V curves [31],[33]. As the heart moves through the contraction cycle it shortens and twists, increasing wall thickness and reducing the cavity volume to expel blood, consistent with experimental results [20],[34]. Table 3 summarises common measures of global heart function from the model and experimental results. Differences between model and experimental pacing frequencies and temperatures limit our ability to compare simulation timing with experimental data.

**Table 3:** Comparison of normal model with end diastolic volume (EDV), end diastolic pressure (EDP), end systolic volume (ESV), end systolic pressure (EDP), endocardium radius (R_endo_), epicardium radius (R_epi_), and end diastolic (ED) and end systolic (ES) wall thickness (WT).

Measure	Model results	Experimental results
EDV (µL)	373.6	397±31 [20], 337±26 [68], 450±90 [33], 440±53 [81], 299±49 [82]
ESV (µL)	129.6	157±18 [20], 172±21 [68], 180±30, [33] 198 [81], 194 ± 47 [82]
EF	65%	61% [20], 49% [68], 60% [33], 55% [81], 35% [82]
EDP (kPa) bracketed numbers in mmHg	1.0 (7.5)	1.19±0.21 (8.9±1.6) [81], 0.44±0.17 (3.33±1.3) [33], 2.0±0.67 (15±5) [82], 1.33±0.27 (10±2 ) [68]
ESP (kPa) bracketed numbers in mmHg	5.0 (37.5)	16.7±0.93 (125±7) [81], 12±2.67 (90±20) [33], 16.9±1.12 (127±9) [82], 12.4±0.80 (93±6) [68]
Diastolic R_endo_ (mm)	3.47	3.8 [81], 3.3 [83], 4.1 [84], 3.9 [20]
Diastolic R_epi_ (mm)	5.44	5.9 [85], 5.8 [20]
Systolic R_endo_ (mm)	2.16	2.3 [81], 2.2 [85], 1.85 [83], 2.45 [84], 2.4 [20]
Systolic R_epi_ (mm)	5.02	5.1 [20]
ED WT (mm)	1.97	1.7–1.9 [81], 1.5–1.6 [86], 3.2 [32], 1.7 [84]
ES WT (mm)	2.85	2.8 [84]

The conductivity tensor was chosen by calculating the propagation velocity of an electrical wave along a model of a rod of cardiac tissue with the approximate dimensions of a papillary muscle (2×2×8 mm), as described previously [13]. A papillary muscle model, as opposed to the left ventricle model was used to validate conduction velocities as the heterogeneous fiber structure in the ventricles complicates comparison between models and experimental results. In the papillary model the activation wave propagation speed was calculated as 0.67 mm ms^−1^. Limited data is available for the conduction velocity in rat cardiac muscle at room temperature. However, 0.67 mm ms^−1^ corresponds to a 26% decrease in conduction velocity from measurements in rat papillary muscle at body temperature (0.9 mm ms^−1^) [35], consistent with decreases of ∼30% observed in rabbit [36],[37] when cooled from 37°C to 27–25°C. In the left ventricle model the time taken for the activation wave to spread from the endocardium to the epicardium is 3 ms, which compares with experimentally measured values of 2.5 ms in rat [38]. The transverse conduction was taken as 10% of the fibre conductivity, consistent with previous studies [39]. Simulations were performed in the papillary model with the fiber transverse direction aligned with the direction of wave propagation. This resulted in an activation wave velocity of 0.25 mm ms^−1^. This compares with values of 0.3 mm ms^−1^ in rat at body temperature [38]. The ratio of the fibre to transverse velocities is 2.68 comparable, to experimental measurements of 2.5±0.4, reported by Roth [39].

**Figure 4:**
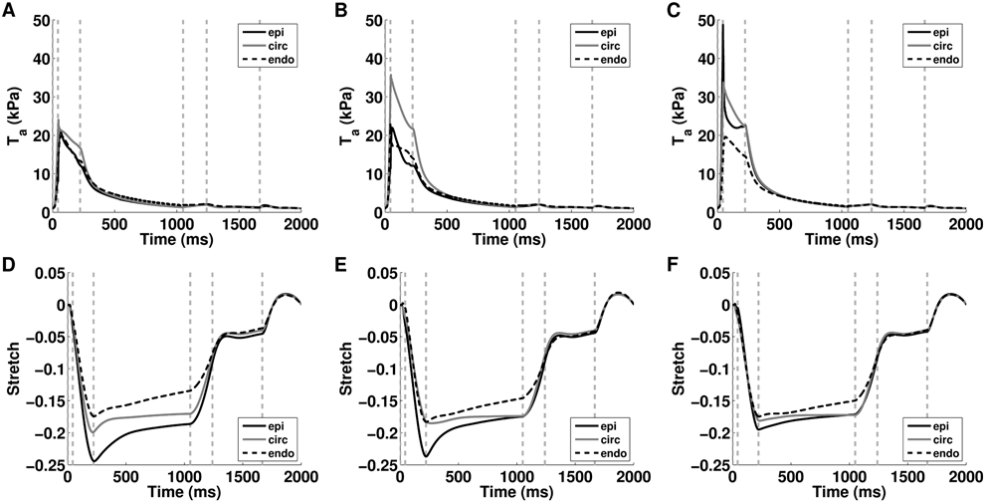
Regional variation in stress and fiber extension ratio in the normal model. Transmural variation in Cauchy active tension at (A) base, (B) mid and (C) apex and extension ratio normalised to end diastolic strain at (D) base, (E) mid and (F) apex from epicardium (epi), midwall circumferential fibers (circ) and endocardium (endo). The gray vertical dashed lines correspond to the transitions in boundary conditions.

Fig. 4A–C and Fig. 4D–E show the transmural fiber strains and active tensions at the apex, mid and base of the ventricle. The strain is normalized to the end-diastolic strain value and the fiber stress is solely the active fiber stress and is distinct from the hydrostatic pressure and passive stress.

### Sensitivity

Central to determining the importance of the TDF mechanisms in defining the ETW is the demonstration that the magnitude of a TDF effect is significant in the context of other experimental and/or individual variations and assumptions in model boundary conditions. For this reason we relate metrics for significant changes to physiological variations in key parameters and boundary conditions. Due to the computational size of the model an exhaustive sensitivity analysis is not tractable. For this reason we have focused on perturbing model parameters/assumptions that are likely to have a significant affect on the spatial distribution of contraction. The removal of a TDF mechanism was considered significant only if it caused a change in function greater than that caused by any of the physiological variations or variations in model assumptions. Table 4 lists the geometrical, mechanical and electrical parameters, boundary conditions and assumption that were tested in the sensitivity analysis.

**Table 4:** Perturbations to heart model used to determine the sensitivity of metrics to model parameters and boundary conditions.

Measure	Change
Fiber angle	varied transmurally from +60° to −60°
Residual strain	±5% variation in residual fiber strains, varied transmurally
EDP	+20%
EDP and ESP	+20% and +20%
ESP	+20%
ESP	−20%
b_o_ (Eq. 1)	+50%
Reference tension and ESP	100 kPa and 10 kPa (75 mmHg)
Beat	1^st^
Heterogeneous electrophysiology	Fast Na^+^ and Ca^2+^ independent transient K^+^ currents varied transmurally
Fiber direction Conductivity	−20%
Location of stimulus current	lower half of endocardium
Duration of stimulus current	5 ms

Different studies have measured different values for the fiber angle in the rat left ventricle [17],[40]. To ensure that the study conclusions were not dependent on the choice of fiber angle, simulations were performed with the fiber angle varying from +60° to −60° from the endocardium to epicardium, as measured experimentally by Chen et al., [40]. The rat left ventricle contains residual strains, which are heterogeneous across the ventricle wall [41],[42]. To test the effect of residual strains on simulation results residual strain was introduced in to the model using a “growth tensor”, as described previously [26]. The residual strain varies from ±5% from the endocardium to the epicardium, which falls within the range of experimental measurements [41],[42].

EDP and ESP were varied by 20%, which is within the range of experimental variation listed in Table 3. The stiffness of the myocardium was increased by increasing b_0_ by 50%, which produced a passive P-V curve that matched data by Omens et al. [16] in Fig. 2. The reference tension (maximum isometric tension at zero strain) in the contraction model [24] is less than previous models [43] and the effect of this in the presence of an increased ESP on results is also tested. As noted above, model analysis is performed on the second beat after the heart has reached a mechanical steady state. The sensitivity of the simulation results to using the first, as opposed to the second, beat is included in the sensitivity analysis.

The electrical properties of the rat heart are heterogeneous. To test the significance of electrical heterogeneity the fast sodium and calcium independent transient outward potassium current channel density were set to be 33% higher and 53.23% lower, respectively, on the endocardium than the epicardium, this approximates the heterogeneous cell models developed by Pandit et al. [22] but excludes transmural variations in gating kinetics, which are not supported by the model architecture. Limited experimental data was available to validate the fiber conductivity, as noted above. To test the role of this parameter on model results a simulation was performed with the fiber conductivity reduced by 20%. In all model simulations the stimulation current was applied over the entire endocardial surface, this provides a simple approximation of the Purkinji fiber system and an unambiguous boundary condition. A simulation was run to test the effect of stimulating from only the lower half of the endocardial surface, an approximation of activation sequence initiated by the embedded Purkinji fiber system. The use of a robust stimulation protocol may have also affected results. To test what effect a 10 ms duration stimulus has on results a simulation was performed with a 5 ms stimulus duration.

**Figure 5:**
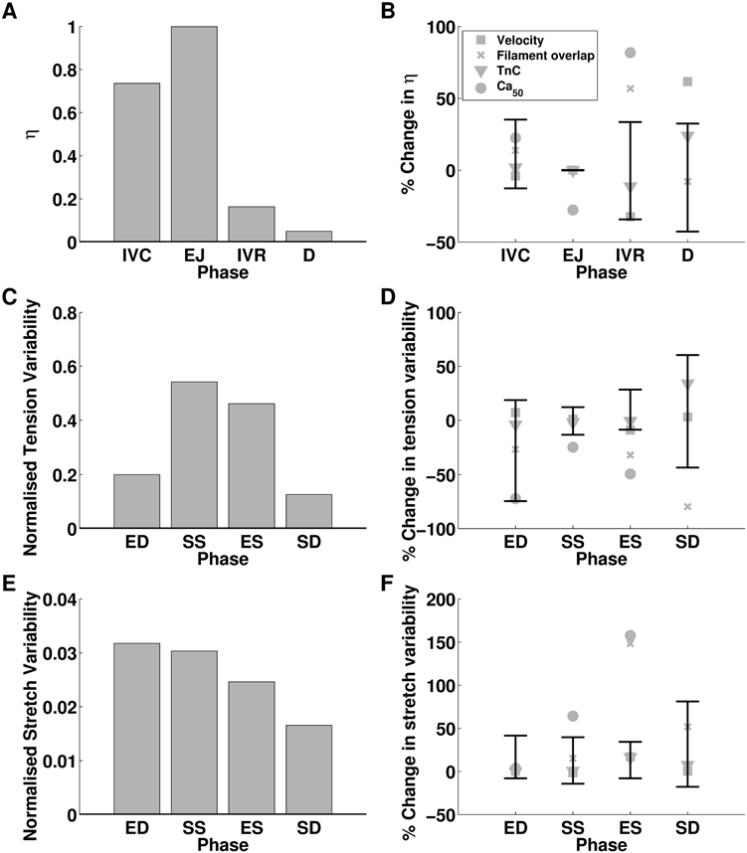
Changes in η and stress and strain homogeneity in the absence of TDF mechanisms. Plots (A), (C) and (E) plot the normal values of η, tension variability and stretch variability, respectively. Plots (B), (D) and (F) plot the relative changes in η, tension variability and stretch variability, respectively, to the normal model, in the absence of each TDF mechanism (velocity dependence of tension (▪), filament overlap (×), tension dependent binding of Ca^2+^ to TnC (▾) and length dependent Ca_50_ (•)) and the maximum and minimum relative sensitivity of each metric to the perturbations listed in Table 4 (black error bars). η is calculated by integrating the ratio of positive work to the sum of positive and negative work (Eq. 6) over each phase of contraction and is plotted for isovolumetric contraction (IVC), ejection (EJ), isovolumetric relaxation (IVR) and diastolic filling (D). Variability in tension and stretch are calculated at a point in time and are calculated at end diastole (ED), start systole (SS), end systole (ES) and start diastole (SD).

**Figure 6:**
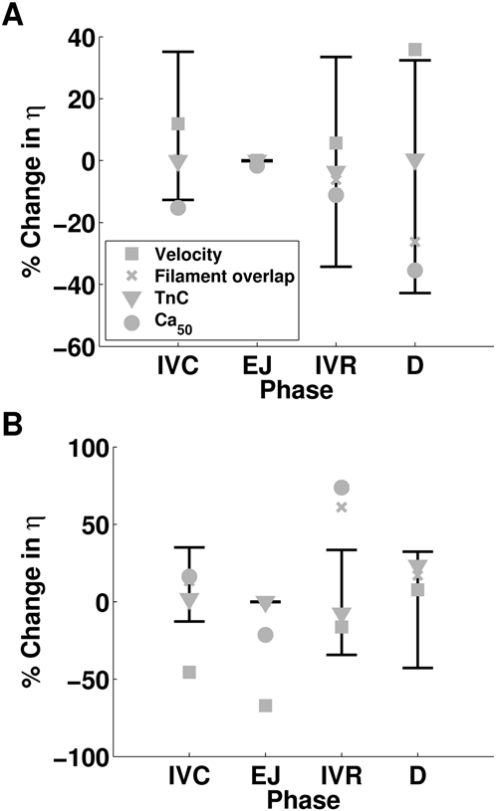
Dependence of changes in η in the absence of TDF mechanisms on the strain and tension fields. Plot (A) compares the percentage change in η in the absence of TDF mechanisms when strain is defined from the normal solution. Plot (B) compares the percentage changes in η in the normal model where strain is defined from solutions in the absence of TDF mechanisms.

The maximum and minimum sensitivity of each function measured are displayed in Figures 5B, 5D and 5F and 6 through the use of error bars. The error bars provide an estimation of the biological and model variability in measurements. In this way the error bars provide a context for analysing changes in model function and any values that fall outside these bars are deemed to have a significant effect.

### Dependence of efficient work transduction on TDF mechanisms

The ETW in the heart was calculated in the model with the default parameter set (normal model), in the presence of all TDF mechanisms. η during each phase of contraction is shown in Fig. 5A. There is a clear change in η values between the contracting (IVC, ejection (EJ)) and relaxing (IVR, diastole (D)) phases in the contraction cycle. With contracting phases tending towards an η value of one with the whole heart contracting, and relaxing phases tending towards an η value of zero with the whole heart relaxing. The value of η during IVC is less than one and reflects the elongation of shorter, but tension generating, regions in the presence of shortening tension generating regions, this regional asynchrony and the corresponding inefficiency in the transduction of work is discussed below. The η value during IVR is higher than in diastole, reflecting a degree of post systolic shortening.

The data points in Fig. 5B show the change in η during each phase in the contraction cycle, calculated in the absence of each TDF mechanism, and the error bars show the sensitivity of η to the parameter and boundary condition perturbations listed in Table 4. Due to the reduced rate of relaxation the results for length dependent Ca_50_ are taken from the first beat and do not include the diastolic period. The effect of this on results is discussed below. During IVC none of the TDF mechanisms caused a significant change in η. The largest change in η during IVC, due to the absence of a TDF mechanism, was caused by the absence of length dependent Ca_50_ (22.38% increase) but this was the same as the change in η caused by increasing ESP by 20% and so was not considered significant. During the ejection phase η varied by less than 0.1% in the sensitivity analysis, described above. Simulations performed in the absence of the filament overlap TDF mechanism caused a 0.8% decrease in the η value during ejection. This is a small decrease but given the low variability in η, in the sensitivity analysis, it is considered significant. The absence of length dependent Ca_50_ has a significant effect on η during ejection, causing a 27.72% decrease in η. This is caused by a fundamental change in the pattern of contraction, as described below. During IVR length dependent Ca_50_ and filament overlap both have significant effects on η, increasing η by 81.69% and 56.74% respectively, compared to the maximum sensitivity analysis increase of 24.41% when both EDP and ESP were increased. The velocity dependence is the only TDF mechanism that causes a significant change in η during diastole (61.53% increase), this compares with the maximum increase of η in the sensitivity analysis, of 32.37% in the presence of a 20% increase in ESP.

### Dependence of tension and strain variability on TDF mechanisms

Fig. 5C and 5E plot the variability in strain and tension at end diastole (ED), start systole (SS), end systole (ES) and start diastole (SD) for the normal heart model. The variability in tension is significantly larger than the variability in strain at each point in the contraction cycle. Variability in tension is also significantly elevated at SS and ES, compared to the slow decrease in variability in strain from ED to SD.

Fig. 5D and 5F display the changes in tension and strain variability, respectively. Points correspond to changes in variability due to the absence of a TDF mechanism and the error bars correspond to the maximum change in variability calculated in the sensitivity analysis. For both tension and strain variability the filament overlap and the length dependence of Ca_50_ have significant effects. In comparing the changes in variability it is notable that significant changes in tension variability are negative and significant changes in strain variability are positive. These changes are consistent with the idea that the TDF mechanisms induce a heterogeneous tension field that results in a homogenous strain field.

In the normal model strain variability decreases as the ventricle model progresses through the phases of the contraction cycle. The absence of the filament overlap and the length dependence of Ca_50_ TDF mechanisms causes the variability to increase between ED and SS, and SS and ES, unlike any of the other simulations performed. This raises the possibility that the absence of one or both of these mechanisms not only perturbs the development of tension at the cellular scale but results in a significantly different contraction pattern at the whole organ scale.

### Dependence of efficient work transduction on stretch

In the heart, strain and tension generation are intimately linked, with strain determining tension development and tension determining deformation and hence strain. In the simulation results presented above, removing the length dependent Ca_50_ and filament overlap caused a significant increase in strain and decrease in tension variability, while at the same time causing a decrease in the ETW. As both the strain and tension fields are dependent on each other it is not clear, based on the previous simulations, through which of these fields the TDF mechanisms act to induce an efficient transduction of work during a contraction. The absence of TDF mechanisms may cause a decrease in the ETW by creating a strain field or a tension field that results in an inefficient contraction. If the strain field calculated from a model solved in the absence of a TDF mechanism causes a decrease in the ETW, even when tension is calculated by the cell model in the presence of all TDF mechanisms, then it is the strain field that compromises the ETW. Alternately, if the strain field calculated from the normal heart model results in a compromised ETW when tension is calculated from a cell model in the absence of a TDF mechanism then it is the tension field that compromises the ETW. To determine which of these is the case, the strain and tension fields were uncoupled.”

In the modelling environment it is possible to save the location of the model nodes at each time step and from this information the strain at each time step can be calculated. It is also possible to read in the node locations and hence define a strain field at each time step. Using these two features it is possible to uncouple strain and tension. First the node positions are saved for simulations of the normal model and in models missing each one of the TDF mechanisms. Secondly the normal model was re-solved, except instead of calculating the node locations at each time step, the node locations were read in from the saved node positions calculated previously in the absence of one of the TDF mechanisms. Similarly, the models in the absence of a TDF mechanism were re-solved, except at each time step the node positions were read in from the solution of the normal model. Fig. 6A plots η for models with a TDF mechanism absent but strain defined from the solution of the normal model and Fig. 6B plots η for the normal model, where strain is defined from the solution of the model with an absent TDF mechanism. In both plots the error bars indicate the sensitivity of η to the changes listed in Table 4 for the fully coupled case. In Fig. 6A, where strain is defined from the normal solution and tension is calculated in the absence of TDF mechanisms there are only nominal changes in η. This compares with the significant change in η observed in Fig. 6B, where strain is calculated in the absence of a TDF mechanism and tension is calculated from the normal model. Thus the significant changes in η observed in the absence of length dependent Ca_50_ and filament overlap are a result of the strain field induced in the absence of each mechanism and not the tension field.

### The efficiency of work transduction and regional work

**Figure 7:**
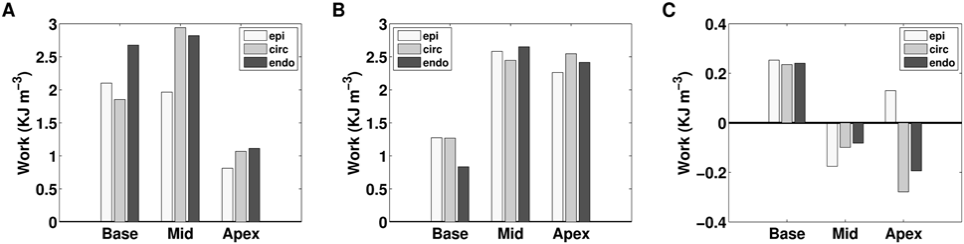
Comparison of regional work in the normal model with simulations performed in the absence of length dependent filament overlap or length dependent Ca_50_. Transmural variation in work density at base, mid ventricle and apex from epicardium (epi), midwall circumferential fibers (circ) and endocardium (endo) for (A) the normal model, (B) in the absent of length dependent filament overlap and (C) in the absence of length dependent Ca_50_.

The absence of the length dependent filament overlap and length dependent Ca_50_ leads to a decrease in the ETW, which coincides with a decrease in synchrony. Previous studies have investigated the relationship between regional work and synchrony and have proposed regional work as an important determinant of perfusion, metabolism, structure and pump function [44]. The transmural variation in regional work was calculated at the apex, mid and base of the heart in the normal model simulations and in simulations in the absence of each of length dependent filament overlap and length dependent Ca_50_ to test if these TDF mechanisms play a role in regulating regional work. The distribution of regional work from these simulations is plotted in Fig. 7.

The absence of length dependent Ca_50_ significantly increases the heterogeneity of work and in the some cases the work is performed on the fibers (negative work), as opposed to the fibers performing work. These regions that perform a net negative amount of work over a contraction cycle are likely to significantly decrease the ETW for the whole ventricle. Hence there presence in the absence of length dependent Ca_50_ is consistent with this TDF mechanism regulating the ETW.

The absence of length dependent filament overlap changes the distribution of work. However, the spatial variation is significantly closer to the normal case than the change in regional work in the absence of length dependent Ca_50_. The absence of length dependent filament overlap results in the base performing less work and the apex performing more than the normal case, however, regional work does not account for the increased volume of the base. Thus, a change in regional work at the base has a greater effect than a comparable change at the apex. This difference between point wise and integral measures is likely to explain the relatively small change in the regional work compared to the significant changes in η caused by the absence of length dependent filament overlap.

### The source of impaired work transduction

**Figure 8:**
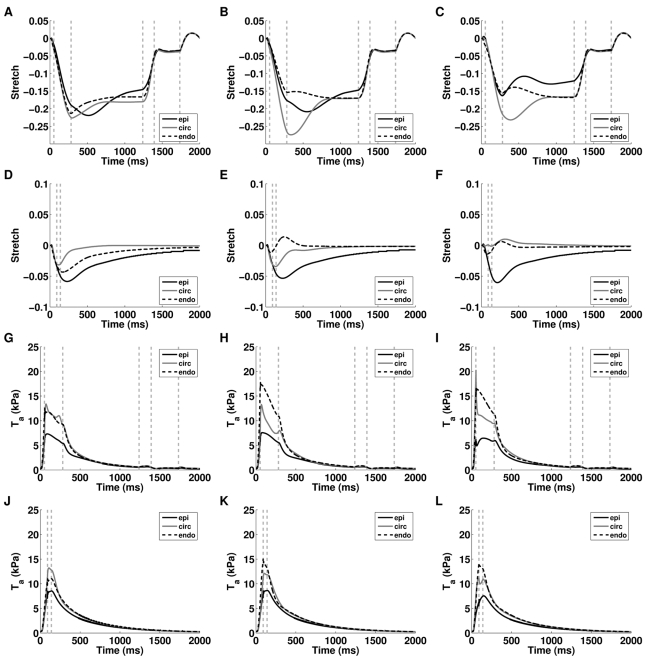
Regional variation in stress and fiber extension ratio for simulations performed in the absence of length dependent filament overlap or length dependent Ca_50_. Transmural variation in Cauchy active tension at the base, mid and apex from simulations in the absence of length dependent filament overlap plots (A), (B), and (C), respectively and length dependent Ca_50_ plots (D), (E), and (F), respectively from the epicardium (epi), midwall circumferential fibers (circ) and endocardium (endo). The extension ratio normalised to end diastolic strain at base, mid and apex from simulations in the absence of length dependent filament overlap plots (G), (H), and (I), respectively, and length dependent Ca_50_ plots (J), (K) and (L), respectively, from the epicardium (epi), midwall circumferential fibers (circ) and endocardium (endo). The gray vertical dashed lines correspond to the transitions in boundary conditions. In the absence of length dependent Ca_50_ the model does not reach diastole, this reduces the number of vertical gray dashed lines.

The previous results predict that the length dependent filament overlap and the length dependence of Ca_50_ are the dominant regulators of the ETW from the cell to the whole organ (Fig. 5B) and the efficiency is primarily determined by the strain (Fig. 6B) as opposed to the active tension fields (Fig. 6A). The metrics of work transduction and homogeneity analysed above show qualitatively similar changes in the absence of these two TDF mechanisms but these metrics are volume integrated and thus obfuscate potential differences between these TDF mechanisms. Calculations of regional work (Fig. 7), shown above, demonstrate that there are significant differences in the role of length dependent Ca_50_ (Fig. 7C) and the filament overlap in regulating regional work (Fig. 7B). Fig. 8 shows how these point variations in regional work are manifested in regional strain (Fig. 8A–F) and stress (Fig. 8G–L). Comparing the transmural variation in extension ratio in the normal model at the base (Fig. 4A), mid (Fig. 4B) and apex (Fig.4C) of the ventricle with corresponding extension ratios in the absence of length dependent filament overlap (Fig. 8A–C) and in the absence of length dependent Ca_50_ (Fig. 8D–F), shows that the absence of either TDF mechanism introduces increased asynchrony in the strain patterns. In particular, the absence of length dependent Ca_50_ from the model predicts significant regions of elongating myocardium even during ejection, as shown by the endocardium extension ratio (dashed line) transients in Fig. 8E and 8F.

**Figure 9:**
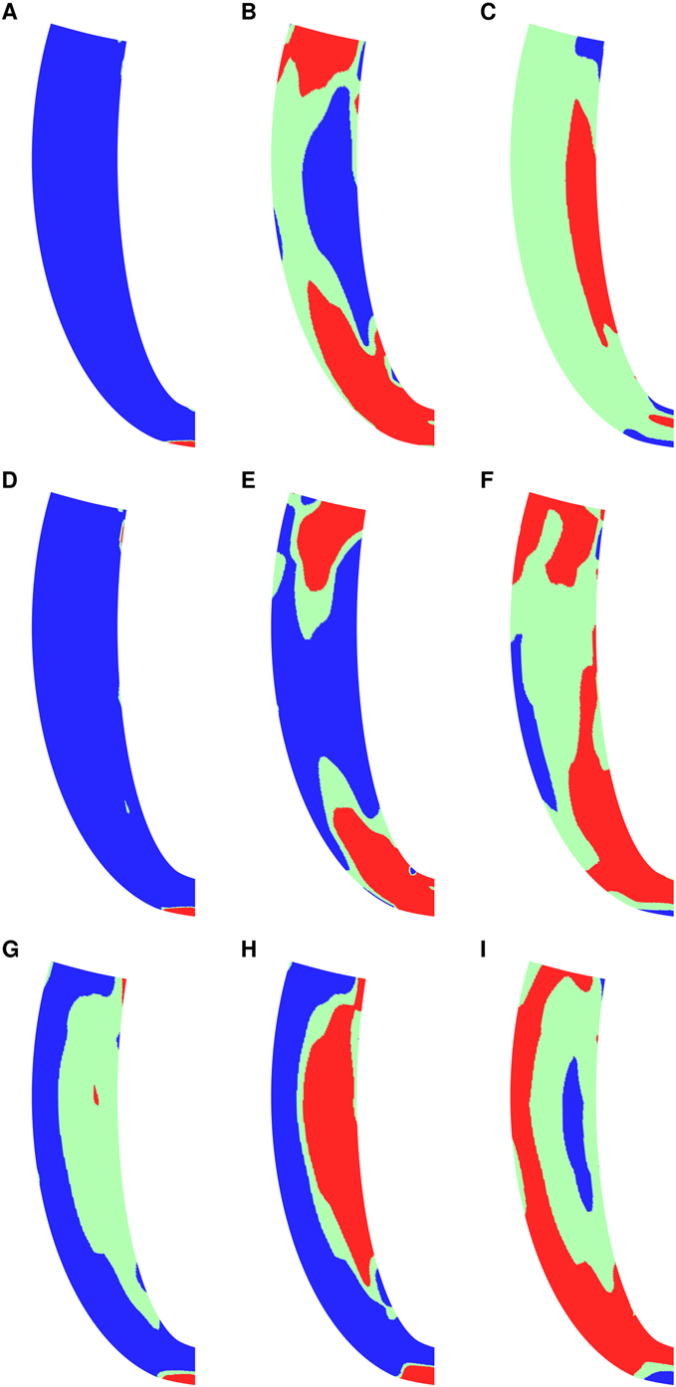
Velocity maps for heart long axis for the normal heart (A–C), in the absence of filament overlap (D–F) and in the absence of Ca_50_ (G–I). With row one ((A), (D), and (G)) representing end IVC, row two ((B), (E), and (H)), representing end ejection and row three ((C), (F), and (I)) representing mid relaxation (400 ms from end diastole). Red regions are elongating, blue shortening and green are static.

To unravel the differences between these two mechanisms the spatial aspects of contraction are now analysed. The fiber strain velocity field was used to characterize the differences in positive and negative work in the two cases, as tension is always positive. The fiber strain velocity was calculated as the difference in the fiber strain field between two consecutive deformation solutions. As the heart model is symmetrical, the long axis cross section of the fiber strain velocity field shows the variation of fiber strain velocity across the whole ventricle. Fig. 9 compares the fiber strain velocity in the long axis cross section of the ventricle, where blue is contraction, green static and red elongation. In the normal heart at end IVC the whole heart is contracting (Fig. 9A), corresponding to the η value of one. At end ejection a significant portion of the heart, in the apical and basal regions, are elongating (Fig. 9B) and at 400 ms (mid IVR) the heart is largely static, with elongation at the mid endocardium (Fig. 9C). The absence of length dependent filament overlap shifts this pattern during IVR, where fewer regions are elongating at end ejection (Fig. 9E) and regions of the epicardium are still contracting after 400 ms (Fig. 9F). Unlike the absence of length dependent filament overlap the absence of length dependent Ca_50_ causes a complete change in the contraction pattern. At end IVC large portions of the heart are not contracting (Fig. 9G), by end ejection the mid endocardium regions are elongating while the apical and basal regions are contracting (Fig. 9G), the opposite of the normal case, and after 400 ms the mid myocardium and sub endocardium regions are contracting (Fig. 9I), again the opposite of the normal case.

## Discussion

In the heart ETW is the result of a complex network of feedback mechanisms and compounding factors. To begin to isolate the regulators of ETW in the heart we have developed a multi-scale biophysical model of the rat heart. The model represents the subcellular contractile mechanisms, Ca^2+^ subsystem, electrophysiology, passive material properties, anisotropic fiber structure and left ventricle geometry spanning the mm to µm spatial- and ms to s temporal- scales. In the absence of a compatible metric of efficiency of work transduction we have defined η, a measure of the ratio of positive to total work and normalized variability measures of tension and stretch to quantify the homogeneity of the model. The model uses strong coupling between the electrophysiology and mechanics models to explicitly represent the links between these two systems. We have solved the model in the absence of each of the TDF mechanisms where strain was calculated using the equations of finite elasticity or defined using the calculated strain field of the normal model and in the normal model with all TDF mechanisms turned on with strain calculated using the finite elasticity equations or defined as the strain field calculated in the absence of each of the TDF mechanisms. We have tested the sensitivity of the model to each of the boundary condition parameters and many of the model assumption, to provide a context for changes in the η and homogeneity metrics following the removal of a TDF mechanism.

### Strong electromechanics coupling

The left ventricle model couples electrophysiology and mechanics using a strong coupling method [13], whereby the mechanics and electrophysiology models are solved at the same time allowing the explicit representation of mechanical feedback on electrophysiology. Specifically, it allows the representation of tension dependent buffering of Ca^2+^ via tension dependent binding of Ca^2+^ to TnC. Furthermore, the addition of strong coupling allows the effects of heterogeneous electrical properties on contraction and hence η to be tested. Although the model results do not show a significant effect of tension dependent binding of Ca^2+^ on synchrony or variability it is only by the explicit inclusion of this mechanism that this conclusion can be drawn.

### Normal model results

The model replicates a wide range of experimental data as demonstrated by Table 3. However, experimental measures of the distribution of stress and strain (see Fig. 4) vary across the literature and are not all consistent with the model predictions.

Consistent with previous modelling [45] and experimental [46]–[53] results the model predicts only small variations in the ES fiber strain with all values falling within 3.5% of −21% (see Fig. 4). The model predicts moderately shorter fiber strains in the endocardium consistent with some [49],[50], although not all [54] experimental measures. The difference between experimental and model results are potentially due to species and fiber orientation differences that can greatly alter fiber strain measurements [49]. The change in fiber strains during relaxation are comparable to experimental results. In the model the endocardial and epicardial fiber strains decrease by 0.038 and 0.0675 compared with 0.031 and 0.064 [48] in the canine heart. In rat hearts Omens et al. [55] found fiber strain to be smaller in the circumferential fibers compared to the epicardium but the results were obtained from two separate preparations and in the circumferential fiber experiments systolic pressure was lower, potentially accounting for the drop in strain. A common observation in experimental measures of strain in the heart is a decrease in circumferential strain from endocardium to epicardium [46], [49], [52], [54], [56]–[58], which is replicated by the model with the peak Green strain decreasing from −0.07 to −0.01 from endocardium to epicardium.

The active stress profiles (see Fig. 4) are qualitatively consistent with circumferential stresses calculated by Holmes [59]. The endocardial stress was smaller than epicardial stress, whereas endocardial fiber stress is often calculated to be higher than epicardial fiber stress [60],[61], however, in these cases the stress value included hydrostatic, passive and active fiber stress. Including the hydrostatic pressure term in the model results in an addition of 5 kPa and 0 kPa to the endocardial and epicardial fiber stress respectively, which results in an endocardial stress higher than the epicardial. The stresses typically increase from apex to base, in the opposite to some experimental observations [62] but nominal apical-basal variation in circumferential stress is also reported, when the curvature of the heart is taken into consideration in the stress calculation [63]. The heterogeneous distribution of active tension predicted by our model generates a heterogeneous work due to the presence of uniform strain. Experimental measures of blood flow and metabolism, which have been shown to correlate with work in isolated preparations [64] and in the whole heart [65], are also heterogeneous across the heart [66], consistent with model predictions.

The end systolic pressure predicted by the model is considerably less than the experimental results listed in Table 3. This is potentially a result of the difference in the model simulation and experimental conditions or the choice of model parameters. The experimental data is acquired at 37°C at physiological pacing frequencies (5–7 Hz) and achieve ESP values of 12–16.7 kPa (90–125 mmHg). This compares to the model which aims to replicate the rat left ventricle at room temperature (20–25°C) at 0.5 Hz and achieves an ESP value of 5 kPa (37.5 mmHg). Hypothermia studies in rat that compare ESP at body temperature and 13°C find a significant decrease in ESP of 70% from 18.7 to 5.5 kPa (140 mmHg to 41 mmHg) [67]. Although 13°C is less than the models temperature (20–22°C), the model ESP is still expected to be significantly less than the ESP values recorded at physiological temperatures, however, the ESP predicted by the model is still lower than expected.

It is possible to achieve a higher ESP by altering the parameters in the model of contraction. As demonstrated in the sensitivity analysis, increasing the parameter describing the reference tension (maximum isometric tension at zero strain) allows the model to reach ESP values of 10 kPa (75 mmHg) that are with in the range of experimental results [33],[68]. This reference tension and ESP combination were included in the sensitivity analysis. All significant changes in the metrics of efficiency and homogeneity fell outside the maximum and minimum changes calculated in the sensitivity analysis. This result reduces the possibility that the study conclusions were dependent on boundary conditions, model parameters or model assumptions.

### The efficiency of the transduction of work from cell to pump function

#### Normal heart

In the case of the normal heart beat the model predicts that strain variability is reduced during contraction, while the variability in tension is increased (Fig. 5C and 5E). The heterogeneous tension field combines with the homogenized strain field to cause a heterogeneous work field. Fig. 5A shows the ETW in each phase of the contraction cycle. For an efficient contraction the sarcomeres need to shorten while generating tension and elongate while generating nominal tension in time with the phases of the heart. The model predicts regions of negative work during IVC (0<η<1), corresponding to tension generating regions elongating. Elongation takes place over the first half of IVC peaking with 45% of the heart elongating 14 ms after activation. Although the velocity of elongation may be small in some regions, at 23 ms the whole heart is contracting and IVC ends at 43 ms (see Fig. 7A). This elongation during IVC also corresponds to a period of reduced strain variability as longer myocytes generate more tension to elongate shorter myocytes. The average length of a shortening region is ≈8% larger than the length of the elongating regions. This pre-stretch is reported in the healthy canine [49],[69],[70], porcine [57], guinea pig [71] and human [4] hearts but the role of the Frank-Starling Law on pre-stretch is debated [4],[69].

During ejection, when the heart is contracting, maximizing positive work is optimal for efficiency, which is seen in the model (η≈1) regardless of perturbations in model boundary conditions (see Fig. 5D), reflecting the robustness of contraction during ejection.

During IVR the heart is relaxing and any positive work will not be transferred to kinetic energy in the blood but will be dissipated through negative work by the myocardium. Therefore a cell contracting during IVR will elongate another cell but not result in any further ejection or effective external work. The normal model exhibits some degree of post systolic shortening during IVR as represented by η>0, consistent with experimental results [57],[70],[72],[73].

In the model, the venous blood pressure is assumed to be 0.7 kPa (5.25 mmHg). Upon reaching 0.25 kPa (1.88 mmHg) during IVR the heart has induced a negative pressure gradient across the mitral valve. At the beginning of diastole there is a increase in pressure within the ventricle (from 0.25 kPa (1.88 mmHg) to 0.7 kPa (5.25 mmHg)) as blood flows into the ventricle rebalancing the pressure gradient. After a period of rest the atrium contracts priming the ventricle before contraction. During this complex phase of events the heart's volume, and hence sarcomere lengths are driven by external forces, by blood returning and the pressure wave associated with atrial contraction. This results in both increases and decreases in volume and sarcomere length during diastole, resulting in a small η value. The external factors and complex volume changes mean that it is hard to compare the diastolic period with experimental results, however η is small compared to all other phases, indicating that the myocardium is in a fully relaxed state. This is consistent with the assumption that the heart is tuned for an efficient contraction.

#### TDF mechanisms

The role of TDF in regulating the ETW was investigated in three ways. Firstly each mechanism was removed and the effect on η was recorded. Secondly, each mechanism was removed but the strain field fed into the cellular model was defined to be the strain field calculated using the normal model at the same time point. Thirdly, the normal model was solved with the strain field defined by the solution to the model in the absence of each TDF mechanism. In solving the model in three ways we can separate the effect of the strain field and tension development on the ETW.

In the absence of length dependent Ca_50_, η during ejection is significantly different from the normal model. The model predicts that the absence of length dependent Ca_50_ causes the mid circumferential and endocardial fibers to generate insufficient tension to contract as the heart shortens in the apex-base direction during ejection. In the normal heart the circumferential fibers have the highest strain, generate the most tension and perform the most work accordingly (see Figs. 4 and 7A). In the absence of length dependent Ca_50_ the heart shortens in the apex-base direction from the contraction of the epicardial fibers but the wall does not thicken, with R_endo_ increasing by 2% as opposed to decreasing by 38% during ejection, resulting in an increase in circumferential fiber strain. In the absence of length dependent Ca_50_, increasing the circumferential fiber strain increases tension but insufficiently to limit this mid wall radial deformation. Thus despite generating more tension the stretched circumferential fibers elongate during ejection, thus partially decoupling strain and tension and resulting in negative work in the mid ventricle circumferential fibers (Fig. 7B). This asynchrony and the resulting impaired transduction of work during contraction is primarily the result of the strain field as efficient work transduction is maintained over ejection in the absence of length dependent Ca_50_ with the strain field prescribed from the solution of the normal model (Fig. 6A). However, when the normal model is solved with the strain field calculated in the absence of length dependent Ca_50_ contraction the transduction of work is significantly impaired (Fig. 6B).

During IVR the absence of either length dependent Ca_50_ or filament overlap causes a significant decrease in the efficiency of work transduction (Fig. 5B). The primary cause of this inefficiency in IVR in the absence of filament overlap effect is the decreased fraction of the heart relaxing at end ejection (Fig. 9H). In the normal heart 41% of the heart is already elongating when IVR begins, compared to 27% in the absence of filament overlap effects, this gives rise to increased post systolic shortening. Thus, filament overlap facilitates a synchronous and efficient end of contraction. In the absence of the filament overlap TDF mechanism a greater fraction of the heart continues to contract during IVR when the work performed by shortening fibers can not be converted into pump function, thus decreasing the ETW. In the absence of length dependent Ca_50_, 38% of the heart is elongating at ES, comparable to the normal heart. However, the spatial distribution of strain (Figs. 5F and 8D–F), stress (Figs. 5D and 8J–L), work (Fig. 7C) and velocity (Fig. 9G–I) are significantly different in the absence of length dependent Ca_50_ compared to the normal model strain (Figs. 5C and 4D–F), stress (Figs. 5E and 4A–C), work (Fig. 7A) and velocity (Fig. 9A–C) and the strain (Figs. 5F and 8A–C), stress (Figs. 5D and 8G–I), work (Fig. 7B) and velocity (Fig. 9D–F) in simulations in the absence of length dependent filament overlap. During ejection, as mentioned above, the mid circumferential fibers are elongated, causing negative work during ejection. A result of this is that during relaxation these fibers shorten. It is the shortening of these tension generating fibers during relaxation that causes increased η, and hence decreased efficiency, during IVR in the absence of length dependent Ca_50_. Again for both filament overlap and length dependent Ca_50_ the change in η during relaxation is primarily a result of the strain field as demonstrated in Fig. 6B.

The absence of the velocity dependence of tension caused a significant increase in η during diastole. This is attributed to the large deformations that occur during diastole and hence primarily affect the velocity feedback mechanism. As the strain field is the primary determinant of the ETW and during diastole the external loading and not the cell contraction govern deformation, the velocity dependence of tension development was not considered to have a major role in determining the ETW in the heart.

#### The joint role of structure and TDF mechanisms

Figs. 7B and 7C and 8 show that despite similar patterns in the change in η and homogeneity (Fig. 5B, 5D, and E), the absence of length dependent filament overlap and Ca_50_ cause significant changes in the local stress, strain and work. Fig. 9G–I predicts that the length dependent Ca_50_ sensitivity plays an important role in cardiac contraction, not only regulating the ETW but defining the spatial pattern of contraction in the heart. The absence of length dependent Ca_50_ sensitivity results in the elongation of the mid myocardium circumferential and endocardial fibers. Without the contraction of the circumferential fibers the heart does not contract in the radial direction and wall thickening is not observed, greatly reducing the ejection fraction.

The elongation of fibers during ejection decreases the synchrony during ejection and this asynchrony during ejection results in elongated fibers at the start of IVR that must shorten to relax, resulting in decreased synchrony of relaxation. This chain of events causes inefficiencies in the transduction of work over each phase of the contraction cycle, resulting in the decreased η during contraction, increased η during relaxation and a significant increase in the heterogeneity of regional stress, strain and work,

#### The role of electrophysiology in the transduction of work

It is possible that changes in homogeneity and the ETW are due to alterations in electrical activation times and/ or changes in the Ca^2+^ transient due to altered deformation patterns, tension generation and Ca^2+^ buffering. The spread of electrical activation across the heart wall in the normal heart took 3 ms, as noted above. In the absence of length dependent Ca^2+^ sensitivity and length dependent filament overlap the activation time remained constant.

In the normal heart model the Ca^2+^ transient has a peak Ca^2+^ value of ∼1.0 µM , basal Ca^2+^ value of ∼0.065 µM and half activation of ∼130 ms and a max/min SR Ca^2+^ content is ∼0.66/ ∼0.28 mM. In the absence of length dependent Ca^2+^ sensitivity or filament overlap none of the metrics of Ca^2+^ dynamics changed by more than 7%. The activation times and Ca^2+^ dynamics are similar in the presence and absence of length dependent Ca^2+^ sensitivity and filament overlap. This indicates that the changes in homogeneity and ETW, as shown in Fig. 5B, are a result of changes in length dependent tension activation within the myofilaments, as opposed to secondary changes in Ca^2+^ dynamic or electrophysiology.

#### TDF mechanisms and heart failure

In this study the ETW from the cellular scale to the whole organ is found to decrease in the absence of the Frank-Starling Law mechanisms. This result may have important implications in the study of heart failure. In the failing myocardium the Frank Starling Law is impaired [74], this coincides with an increase in asynchrony of contraction [75] and a decrease in efficiency [76]. The results of this study provide a potential common cellular scale mechanism for linking these organ scale observations.

### Limitations

The model developed here is invariably an approximation of the cardiac system and as such is subject to limitations based on the knowledge it encapsulates. Although the model is complex it still fails to represent many of the spatial heterogeneities present in the heart, simulations are not performed at true steady states, the end systolic pressure is less than expected and the temperature and pacing frequency of the model are far from physiological. The effects, context and implications of these limitations are discussed below.

To achieve a mechanical steady state all simulations were solved for two beats and the second beat was used for analysis. However, in the absence of length dependent Ca_50_ the model failed to relax sufficiently during IVR to reach diastole. As no second beat was solved all comparisons were performed on the first beat of the absent Ca_50_ simulations. To test what effect solving the model twice has on simulation metrics, the first beat of the normal model is included in the sensitivity analysis, as noted above. The inclusion of these results caused no significant change in the results and hence the use of the first beat from Ca_50_ simulations is unlikely to alter the conclusions of this study.

The model is currently solved to a mechanical steady state, where the P-V curve returns to its starting position by the end of a beat (see Fig. 3C). Although the cell model is paced to steady state in a single cell simulation, prior to its inclusion in the whole organ model, it is unlikely that the cell models within the left ventricle model have reached an electrophysiology steady state after two beats. Given the time required to integrate one beat (50 hours), the maximum number of consecutive beats solved was 6, over this time scale no slow changes in [Na^+^]_i_ were simulated. However, simulations in single cells show that the Ca^2+^ transient reaches a steady state within 5–6 beats, whereas [Na^+^]_i_ can take 100 to 1000 s of beats to reach a steady state following a perturbation. It is possible that the lack of a true steady state dampens the role of tension dependent binding of Ca^2+^ to TnC on the synchrony of contraction, as over time its role may increase by altering Ca^2+^ dynamics via changes in [Na^+^]_i_ and [Ca^2+^]_i_ regulation.

The model ESP was chosen as 5 kPa (37.5 mmHg). This value is significantly less than physiological pressures (see Table 3). The ESP was chosen to achieve optimum work, as calculated by the area enclosed by the pressure volume curve (see Fig. 3C). The ESP chosen resulted in a high ejection fraction, relative to experimental measures, without altering any of the electromechanical cell model parameters. It is possible to manipulate the ESP by adjusting the reference tension in the model of contraction, as demonstrated in the sensitivity analysis, by increasing the reference tension and ESP. We chose not to adjust the reference tension as there was insufficient experimental evidence to indicate that this was the correct parameter to manipulate. However, to confirm that the study conclusions were not dependent on the ESP value, simulations in the absence of filament overlap and length dependent Ca_50_ were performed where the reference tension was increased to 100 kPa and 200 kPa, respectively. The reference tension was set higher in the absence of length dependent Ca_50_ to allow both simulations to reach an ESP of 10 kPa (75 mmHg). Similar changes in the value of η, stretch variability, tension variability and regional stress and strain patterns were found in both models at higher ESP and reference tensions, confirming that the study results are not likely to be dependent on the reference tension and ESP.

The model replicates the rat left ventricle at room temperature. The model parameters were fit to experimental data taken primarily from room temperature as opposed to body temperature. This allowed the model to be both species and temperature consistent and allowed the use of the significant body of experimental data in the literature to link each parameter with experimental data and validate the model. By creating a temperature and species consistent model we have been able to validate the model components across the multiple spatial scales pertinent to this study against an existing physical system, as opposed to species inconsistent models, which face challenges reconciling the intrinsic differences between species. Contraction data is primarily recorded at room temperature [24] and this dictated the temperatures of the subsequent models. Until recently only limited experimental data was available to characterize contraction at higher temperature although recent work at body temperature may remove this limitation in the future [77]. However, to develop comprehensive models of contraction, extensive data sets are required which are not presently available. It is not possible at present to reliably perform the simulations presented here at physiological temperatures with any confidence, due to the inability to validate all of the sub cellular model components. However, the model replicates changes in volume and wall thickness observed in the physiological heart and Fig. 6B demonstrates that it is the deformation field that is a primary determinant of η. Thus the conclusions drawn from this study maintain a relevance for physiological conditions.

The metric of synchrony, η, developed in this paper provides an indirect metric for the chemical energy consumed by the cell to perform external work. Ideally the efficiency by which the heart converts energy in the form of ATP to external work would have been calculated directly, however coupled metabolic- mechanics - electrophysiology models are in their infancy and insufficient experimental data is available to fully characterize the model parameters for a consistent species and temperature. For these reasons we have made no attempt to simulate ATP consumption or production, and have resorted to the implicit assumption that energy consumption is proportional to work, regardless of the direction of contraction. Although this may appear as an oversimplification, experimentally the consumption of oxygen by isolated trabeculae [64] cardiac tissue [65] and the whole heart [78] has been shown to correlate with work using the pressure - volume area or force length area relationship described by Suga [78]. In this study as comparisons are made between model and phases of the contraction cycle using a consistent and rational metric, we do not feel that this approximation alters the study's conclusions.

The definition of η assumes that negative work and positive work consume the same amount of energy. In skeletal muscle the metabolic cost of negative work is nominal relative to positive work [79], however, in the heart, whole organ data predicts that negative work consumes only 27% less oxygen than positive work [80]. This difference may be due to the different roles of skeletal and cardiac muscle under physiological conditions, where skeletal muscle can act as a break, where as cardiac muscle never fulfils this role. Additional experimental measurements, ideally in isolated muscle preparations, are required to fully characterise the energy consumption of positive and negative work in cardiac muscle. If the ratio of energy consumption for negative work to positive work is χ, then η can be refined as W_pos_/(W_pos_+χW_neg_). So long as χ>0, it can be shown that the qualitative relationships in Figs. 5B and 6 will remain true, thus the ratio of energy consumption for negative work to positive work is unlikely to have an effect on the study conclusions.

### Conclusion

This model demonstrates the first three dimensional, biophysical strongly coupled electro-mechanics model of the rat heart. The model is validated against volume, pressure and wall thickness measurements from beating hearts. The model demonstrates that TDF mechanisms play a significant role in regulating the ETW. Specifically the Frank-Starling subset of the TDF mechanisms (filament overlap and length dependent Ca_50_) are essential in creating a homogenous strain during IVC, ejection and IVR. The length dependent filament facilitates a homogenous and efficient end to contraction and the length dependent Ca_50_ sensitivity regulates the spatial contractile patterns of the heart to induce a homogenous and efficient contraction.
